# Headache associated with cough: a review

**DOI:** 10.1186/1129-2377-14-42

**Published:** 2013-05-20

**Authors:** Ann Cordenier, Willem De Hertogh, Jacques De Keyser, Jan Versijpt

**Affiliations:** 1 Headache Clinic, Department of Neurology, Universitair Ziekenhuis Brussel, Brussel, Belgium; 2 Department of Rehabilitation Sciences and Physiotherapy, Faculty of Medicine and Health Sciences, University of Antwerp, Antwerp, Belgium; 3 Department of Neurology, University Medical Center Groningen, Groningen, The Netherlands; 4 Department of Neurology, Universitair Ziekenhuis Brussel, Laarbeeklaan 101, Brussel, 1090, Belgium

**Keywords:** Cough, Headache, Diagnosis, Treatment

## Abstract

Headache only triggered by coughing is a rather uncommon condition. The aim of the present review is to present an overview of the diagnosis, clinical characteristics, pathophysiology and treatment of both primary and symptomatic cough headache and discuss other relevant headache disorders affected by coughing. The diagnosis of primary cough headache is made when headache is brought on and occurs only in association with coughing, straining or a Valsalva manoeuvre and in the absence of any abnormalities on neuro-imaging. In case an underlying pathology is identified as a cause of the headache, the diagnosis of symptomatic cough headache is made. The vast majority of these patients present with a Chiari malformation type I. Other frequently reported causes include miscellaneous posterior fossa pathology, carotid or vertebrobasilar disease and cerebral aneurysms. Consequently, diagnostic neuroimaging is key in the diagnosis of cough-related headache and guides treatment. Besides primary and symptomatic cough headache, several other both primary and secondary headache disorders exist where coughing acts as a trigger or aggravator of headache symptomatology.

## Review

Cough triggering headache is an uncommon finding. It is characterized as headache triggered by rapid increases in intra-abdominal pressure, caused by coughing, sneezing or straining. The life-time prevalence of cough headache is estimated to be 1% [1]. The prevalence in a headache clinic varies from 0.4% to 1.2% [2,3].

Cough headache can be further subdivided into *primary* and *symptomatic cough headache*. It has first been described in medical literature in 1932 by Tinel [4]. Initially, cough headache was considered as an alarm symptom, until both Symonds and Rooke reported cases of *benign cough headache*, now known as *primary cough headache*[5,6]. Up till now, about 400 cases of primary and 300 cases of symptomatic cough headache have been described in literature. In addition to previous reviews on primary cough headache [7], we review the etiology, clinical features and treatment strategies for both primary as well as symptomatic cough headache and shed light on some pathophysiological mechanisms. Moreover, other both primary and secondary headache disorders which are triggered or aggravated by coughing are described.

## Methods

### Search strategy and selection of articles

The MEDLINE database was searched between 1950 up till 2011, using the MeSH terms *“cough”, “headache”, and “Valsava manoever”*. The search was limited to English studies in humans. Articles were included when dealing with diagnostics or therapy of cough-related headache in adults. Articles were excluded when ‘headache’ and ‘cough’ where mentioned as symptoms of other medical conditions (e.g. cold, hypertension, non-specific health symptoms, related to surgical procedures such as stereotactic surgery, or related to substances). Case series and single cases were included. Bibliographies of selected articles were screened for additional relevant articles.

## Cough headache

### Primary cough headache

In 1956, Symonds was the first to describe primary cough headache as a separate disease entity. He described 27 cases of headache provoked by Valsalva maneuvers like coughing, sneezing, straining, laughing or stooping [5]. In 21 of these patients, no intracranial lesion by means of computed tomography could be demonstrated. Later, a series of 93 patients with ‘benign exertional headache’ was described by Rooke in 1968. He did not make a difference between cough headache and headache provoked by physical exercise [6].

Primary cough headache, previously also called *benign cough headache*[7] or *Valsalva-manoeuvre headache*, is currently defined by the International Headache Society (IHS) as a headache, precipitated by coughing or straining in the absence of any intracranial disorder lasting up to 30 minutes [8].

### Diagnostic criteria for primary cough headache (group 4.2 - International classification of headache disorders, 2nd edition, 2004)

A. Headache fulfilling criteria B and C

B. Sudden onset, lasting from one second to 30 minutes

C. Brought on by and occurring only in association with coughing, straining and/or Valsalva manoeuvre

D. Not attributed to another disorder

Table 1 gives an overview of the published case series of primary cough headache.

**Table 1:** Major characteristics of published series on primary cough headache

**First author, Year [Reference]**	**Nr of Cases**	**Patient characteristics**		**Headache characteristics**						
		**M/F ratio**	**Mean age**	**Intensity & type**	**Other triggers**	**Duration**	**Location**	**Frequency**	**Persistence**	**Associated features**
Symonds, 1956 [5]	21	18/3	55	severe bursting	Valsalva maneuver, head rotation	2’-10’	bilateral	-	18 months- 3 years	-
Pascual,1996 [13]	13	10/3	67	moderate to severe sharp, stabbing	Valsalva manoeuver	seconds to less than 30’	bilateral (92%) unilateral (8%)	one to several daily	2-24 months	none
Ozge, 2005 [9]	20	13/7	45	moderate to severe sharp, stabbing	not mentioned or no other triggers?	1-30’	bilateral (90%) unilateral (10%)	10 days/month	-	nausea (5%) dizziness (10%)
Pascual, 2008 [11]	28	10/18	60	electrical, explosive, pressing or having a mixed nature	sudden postural movements, weight lifting, laughing and defecating	seconds to more than 1’	unilateral (50%) bilateral (39%) occipito-suboccipital (11%)	-	1-42 months	dizziness (14%)
Chen, 2009 [3]	74	54/20	61	mild to severe explosive, dull, pulsatile	straining at stool and bending down	1” - 2 hours	bilateral (67%) unilateral (33%)	-	6-24 months	nausea (10%) vomiting (1%) photophobia (5%) phonophobia (11%)

Primary cough headache is usually bilateral but can be unilateral and has a moderate to severe intensity where the type of pain varies. According to Özge et al., pain was mostly located in the frontotemporal regions but even toothache as the presenting symptom has been described [9,10]. It most often affects men, however, Pascual et al. reported on 28 patients with primary cough headache, of which 18 were women [11]. It usually affects subjects over the age of 40. According to the currently available criteria, the headache should last from one second to 30 minutes, but headaches of a longer lasting duration have been reported. For instance, Chen et al. published a series of 74 primary cough headache patients where the median headache duration was indeed 30 seconds, but in a minority of patients the headache lasted up to 2 hours [3]. Nausea, vomiting, photo- and phonophobia are uncommon [12]. Besides cough, headache was also triggered by other Valsalva maneuvers in most of the studies, but never by physical exercise.

### Symptomatic cough headache

**Figure 1:**
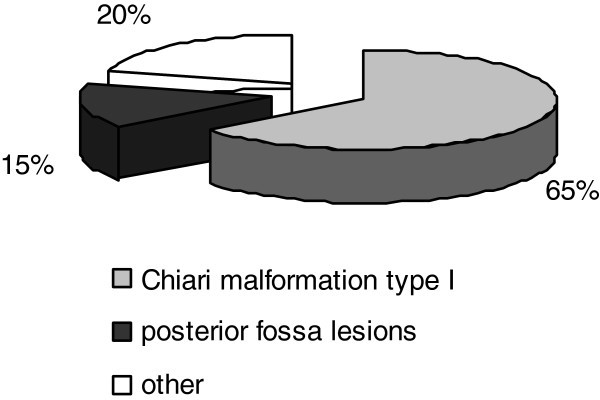
Etiology of symptomatic cough headache.

Underlying etiologies are present in approximately 40% of the cases and are mostly related to Chiari type I malformation. In general, patients with symptomatic cough headache differ from patients with primary cough headache in the fact that they tend to have more associated symptoms, depending on the underlying abnormality. Additional headache triggers, higher pain intensities and diverse headache durations and locations are generally reported. The major causes of symptomatic cough headache are shown in Figure 1. The most common causes are, after Chiari type I malformation, miscellaneous posterior fossa lesions. Other causes include obstructive hydrocephalus and spontaneous low cerebrospinal fluid pressure (CSF).

All of the 17 patients (10 men) with symptomatic cough headache reported by Pascual et al. had a Chiari type I malformation. In contrast to patients with primary cough headache, they all had occipital or suboccipital headache. Pain was described as bursting, stabbing, dull, or lancinating. Headache durations ranged from seconds to several weeks. Besides cough, headache could be provoked by laughing, weight lifting or acute body or head postural changes. All patients also developed one or more posterior fossa symptoms, however, not all from the start [13].

More recently, Pascual et al. reported on forty patients (12 men) with symptomatic cough headache. Thirty-two patients had a Chiari type I malformation. Eight patients had a structural lesion in the posterior fossa: 3 arachnoid cysts, 2 dermoid tumours, 2 meningiomas and 1 os odontoideum. Headache was localized occipito-suboccipital. The nature of the pain was described as pressing, explosive, electrical or having a mixed nature. Duration ranged from seconds to more than one minute. Postural movements, laughing and defecation could also trigger the headache. Thirty-three patients had posterior fossa symptomatology like dizziness, unsteadiness, facial and upper limb numbness, vertigo and syncope [11].

Chen at al. reported on nine symptomatic cough headache patients. The headache profile was similar as in their primary cough headache patient group being mostly bilateral and rarely with associated symptoms. The duration of the headache ranged from 10 seconds to 30 minutes. In this group, headache could be triggered not only by cough, but also by exertion, straining at stool and lifting heavy objects. As underlying causes they found 4 patients with an obstructive hydrocephalus, two patients with a Chiari malformation type I, one patient with a subdural hematoma, one with multiple brain metastases and one with an acute sphenoid sinusitis [3].

Nuti et al. and Evans et al. both described a patient who presented with cough headache due to spontaneous low CSF pressure [14-16]. Mokri also described two patients with headache provoked by cough and Valsalva maneuvers caused by a spontaneous CSF leak [17]. One case of pneumocephalus and pneumococcal meningitis presenting with cough headache was also reported [18]. Eross et al. reported on a case of a 66-year old patient with cough headache where magnetic resonance imaging revealed a posterior fossa mass, associated with obstructive hydrocephalus [19]. Two case reports even mention cough headache as the presenting symptom of carotid artery disease [20,21]. Finally, Smith and Messing report on one case of cough headache associated with a non-ruptured cerebral aneurysm [22].

### Pathophysiology

The pathophysiology of primary cough headache is not well understood, but various hypotheses have been formulated. It seems likely that it is associated with an increased intracranial pressure caused by coughing, this due to an increase in the intra-thoracic and intra-abdominal pressure subsequently leading to an increase in the central venous pressure. A recent study showed a transverse or jugular vein stenosis by means of MR venography in 5 out of 7 patients with primary cough headache, although the debate continues as to whether this stenosis is a primary or secondary process related to raised intracranial pressure [23]. Patients might also have a lower threshold for pain associated with the increase in intracranial pressure caused by coughing [24]. Raskin further hypothesized about the location of hypersensitive pressure receptors on the venous vessel walls [24]. Wang on the other hand proposed that cough headache was caused by CSF hypervolemia, which would lead to an increase in intracranial pressure during coughing [25]. Wolff thought that cough headache was related to a systemic infection, which would alter the vascular tone in the cranial vessels [26]. Finally, Chen et al. found that patients with primary cough headache had a more crowded posterior cranial fossa. This might lead to a relative obstruction of CSF flow, which can contribute to an increase in intracranial pressure during coughing [27].

An increase in intracranial pressure is also believed to be the underlying mechanism of symptomatic cough headache, although the exact mechanism is unknown. In patients with a Chiari malformation type I, this seems to be caused by the sagging of the cerebellar tonsils below the foramen magnum [28,29]. Indeed, Williams described two patients with cough headache and a tonsillar herniation where a difference in pressure between the ventricles and the lumbar subarachnoidal space after performing a Valsalva manoever was demonstrated [28]. This *craniospinal pressure dissociation* displaces the tonsils further into the foramen magnum and pain by coughing could therefore be caused by compression or tracking on pain-sensitive structures in the arachnoid space or blood vessels surrounding the tonsils. This mechanism is supported by the fact that, after surgery, both the *craniospinal pressure dissociation* and cough headache disappear. Moreover, Pascual et al. found that headache correlated with the degree of tonsillar descent [30], although this was not supported by the findings of Sansur et al. They also did not find a *craniospinal pressure dissociation* and postulated that headache was associated with a sudden increase of intracranial pressure, caused by obstruction of free CSF flow in the subarachnoid space [29].

### Treatment

Although no long term studies exist on the natural evolution of cough headache, it seems that most of the primary cough headache patients remit spontaneously after maximum 4 years, however, patients with a disease duration of 12 years and more have been described [5]. Because of the short duration, there is usually no need for an acute treatment. However, since symptoms can be quite debilitating, a preventive treatment strategy should be considered in most if not all patients. Treatment options for primary cough headache are outlined in Table 2. Apart from one small double-blind, placebo-controlled crossover study with indomethacin in a dose of 50 mg tid, no large randomized trials have been performed [31]. General consensus exists that the treatment of choice for primary cough headache is indeed indomethacin, however with varying daily doses, treatment durations and treatment effects with a general response rate of approximately 73% [3]. Several studies found that daily doses ranging from 25–150 mg usually are effective [3,9,11,13,31]. In one study a daily dose up to 250 mg was required [24]. In the series published by Chen et al., less than half of the patients experienced a complete relief where another one third had a partial response [3]. No consensus exists on treatment duration. In the series of Pascual et al., treatment was required for a maximum period of 5 months and in the series published by Chen et al., nearly every patient with a good initial response was pain-free within 6 months after initiation of indomethacin, however, recurrences occurred in a few patients after a minimum interval of 6 months [3,11].

**Table 2:** Reported treatments for primary cough headache

**Product [Reference]**	**Recommended daily dose**	**Most common side effects**
Indomethacin [3]	50-150 mg	peptic ulcers, dyspepsia, edema, hyperkalemia, hypernatremia, hypertension
Topiramate [33]	50-100 mg	cognitive deficits, paresthesia, anorexia
Methysergide [9,34,35]	2 mg	pleuritis, pericarditis, retroperitoneal fibrosis
Acetazolamide [25]	375-2200 mg	paresthesia, parageusia, kidney stones, dehydration, headache, metabolic acidosis
Propranolol [35]	120 mg	hypotension, bradycardia
Naproxen [37]	550-1100 mg	gastrointestinal complaints
Metoclopramide [38]	10 mg intravenous bolus	restlessness, drowsiness, dizziness, fatigue, and focal dystonia

The mechanism by which indomethacin is effective is not fully understood, but indomethacin decreases intracranial pressure which could be the possible mechanism of action [32]. This could also explain why some studies found benefit in treating cough headache with acetazolamide [25] and lumbar punctures [3,24], both known to decrease intracranial pressure. The latter even had a fairly good response rate with 8 out of 10 patients improved in the series published by Chen et al. [3].

Besides indomethacin, beneficial effects of topiramate [33], methysergide [9,34], propranolol [35,36], naproxen [37] and intravenous metoclopramide have been reported [38] in smaller case series.

Patients with symptomatic cough headache usually require a tailor-based surgical treatment. Suboccipital craniectomy, whether or not combined with a C1-C3 laminectomy, relieves cough headache in the majority of patients with a Chiari malformation type I [11,13]. Of interest is the fact that, although not consistently, a response rate to indomethacin of approximately 38% has been described in several symptomatic cough headache patients [3,11,39].

### Other relevant headache disorders, potentially triggered or aggravated by coughing

Next to primary and symptomatic headache, several other both primary and secondary headache disorders exist where coughing is a known trigger for headache symptomatology or where headache can be aggravated by coughing (Table 3). These should therefore be considered in the differential diagnosis.

**Table 3:** Primary and secondary headache disorders provoked or aggravated by coughing

**Disorder**	**Headache quality**	**Cough as trigger**	**(Other) triggers**	**Cough as aggravator**	**(Other) aggravators**
**Primary cough headache**	sharp/stabbing	++	Valsalva	NA	NA
**Symptomatic cough headache**	mixed nature	++	Valsalva	NA	NA
**Idiopathic intracranial hypertension**	non-pulsating	+	Valsalva	+	Valsalva and postural changes
**Headache attributed to intracranial hypertension**	non-pulsating	+	Valsalva	+	Valsalva
**Postictal headache**	pressing/pulsating	-	seizure	+	bending and sudden head movements
**Headache attributed to Chiari malformation**	mixed nature	+	Valsalva	+	NA
**High altitude headache**	dull/pressing	-	> 2500 m	+	exertion, movement, straining and bending
**Migraine**	pulsating	-	see text	+	bending forward, exercise, …
**Tension type headache**	pressing	-	see text	+	fatigue, stress, …
**Cluster headache**	piercing	+	alcohol	NA	NA

NA: not applicable.

#### Headache attributed to intracranial hypertension, idiopathic or secondary

Headache attributed to intracranial hypertension, idiopathic or secondary, is a non-pulsating headache which usually occurs daily and has a moderate intensity. It can worsen by coughing or other Valsalva manoeuvres. It is often accompanied with other abnormalities like papilledema, visual field defects or a sixth nerve palsy [40,41].

#### Post-ictal headache

Post-ictal headache is a tension-type headache or, in a patient with migraine, a migraine headache, appearing after a partial or generalised epileptic seizure [42]. One study of 51 patients with post-ictal headache found that in half of the patients headache could be aggravated by coughing, bending and sudden head movements [43].

#### High-altitude headache

High-altitude headache can appear after an ascent to an altitude above 2500 m. Typical features are onset within 24 hours of reaching a certain height with the appearance of a usually bilateral and dull headache with a duration of less than one day [12,44]. Headache can be aggravated by exertion, movement, straining, coughing or bending [45].

#### Migraine

Migraine is mostly a unilateral headache with a pulsating quality. It is usually associated with nausea and/or photophobia and phonophobia. Physical activity is a well-known aggravating factor [46]. Spierings et al. investigated 38 patients with migraine and reported that patients identified Valsalva-related manoeuvres like straining (87%), bending over (84%) and coughing/sneezing (53%) as aggravating or triggering factors. Other reported triggers or aggravating factors were physical activity, stress, fatigue, reading, driving, lack of sleep, specific foods/drinks, alcohol, not eating on time, smoke, smell, light, noise, menstruation and weather changes [47]. A clear distinction however between factors being a trigger or an aggravator was not made.

#### Tension-type headache

Tension-type headache is considered to be a bilateral pressing headache. As a rule, it is not aggravated by physical activity [48]. Spierings et al. found that tension-type headache could be aggravated by Valsalva-related manoeuvres with 41% of the patients experiencing worsening with straining, 35% with bending over and 29% with coughing or sneezing [47].

#### Cluster headache

Cluster headache is characterised by attacks of a severe, unilateral, orbital, supraorbital or temporal pain, lasting for 15 to 180 minutes. These attacks are associated with ipsilateral conjunctival injection, lacrimation, nasal congestion, rhinorrhea, miosis, ptosis or eyelid edema [49]. Precipitating factors of cluster headache include alcohol, histamine and sublingual nitroglycerine [50,51]. Cases of Valsalva-induced cluster headache are described in which the cluster attacks were only triggered by Valsalva manoeuvres including coughing, sneezing or straining, not by physical exercise [52,53].

## Conclusions

In general, headache triggered by coughing is an unusual clinical symptom which deserves specific attention. The present review describes the clinical characteristics and treatment options of primary and symptomatic cough headache. In addition, various headache disorders which can be aggravated or triggered by coughing were listed. The present overview can guide clinicians in their diagnostic and therapeutic process.

## Competing interests

The authors declare that they have no competing interests.

## Authors’ contributions

AC drafted the manuscript. WDH, JDK and JV provided essential comments to finalize the manuscript. All authors read and approved the final manuscript.
